# Functional representativeness and distinctiveness of reintroduced birds and mammals in Europe

**DOI:** 10.1038/s41598-022-07991-x

**Published:** 2022-03-08

**Authors:** Charles Thévenin, Maud Mouchet, Alexandre Robert, Christian Kerbiriou, François Sarrazin

**Affiliations:** Sorbonne Université, Muséum National d’Histoire Naturelle, CNRS, UMR 7204 Centre d’Ecologie et des Sciences de la Conservation, Muséum National d’Histoire Naturelle, 43, Rue Buffon, 75005 Paris, France

**Keywords:** Restoration ecology, Conservation biology

## Abstract

Reintroduction, the human-mediated movement of organisms to re-establish locally extinct populations, has become a popular conservation tool. However, because reintroductions often focus on local or national conservation issues, their contribution to the conservation of biodiversity at large scale remains unclear. While taxonomic biases have already been identified in reintroduction programs at regional scales, studies have stressed the need to account for other facets of biodiversity when assessing the relevance of the allocation of conservation efforts. In particular, it may be very fruitful to discriminate if and how such taxonomic biases may influence the functional complementarity of reintroduction targets, and to which extent reintroduction practitioners may have focused on species performing more singular functions than others. Here, we investigate the diversity of functional traits supported by reintroduced species of terrestrial birds and mammals in Europe. For each taxonomic group, we explored the functional representativeness of reintroduction targets at the European scale, i.e., whether species involved in reintroduction programs collectively represent the range of functional trait variation observed in the regional assemblage. Because additional conservation value could have been given by practitioners to species performing singular functions, we also measured the functional distinctiveness of reintroduced species. We found that reintroductions of birds did not focus on functionally distinct species, and that the subset of reintroduced birds is representative of the functional diversity at a continental scale. However, reintroductions of mammals involved more functionally distinct species than expected, even though reintroduced mammals are not collectively representative of the functional diversity of the continental assemblage.

## Introduction

How species diversity contributes to ecosystem functioning is one of the core debates in ecology^1,2^, and one critical issue is the extent to which ecosystem functioning is buffered against species loss^3–7^. In ecosystems with high levels of functional redundancy, i.e. the fact that several species can support the same function^8^, it was assumed that a high proportion of species could be lost before inducing the disappearance of functional groups in natural communities^9^. However this assumption has been challenged, even in species rich systems where many functions are left highly vulnerable and supported by a few species only^10^.

One way to indirectly assess the individual roles of species in assemblages is by studying functional trait diversity. Functional traits are any measurable morphological, behavioural or phenological features of an organism, which relate to ecological processes that can potentially influence fitness and performance^11^. Trait based-approaches can be used to address a variety of ecological questions^12,13^, and provide a way to measure functional diversity by summarizing the variation in trait values between organisms^6,14^. Functional diversity is a multi-faceted concept that can be considered at multiple ecological scales, from populations and communities to regions and continents^15^. Because of its great potential to describe ecological processes, the characterization of functional diversity provides a compelling framework to evaluate the potential consequences of conservation strategies. For example, one assumption of this trait-based approach is that species with more distinct combinations of functional traits are more likely to support functions that may not be delivered by species with more common associations of traits^2,16^. Furthermore, highly distinct combinations of traits seem to be supported by rare species, thus the functions they support might be more vulnerable to extinction^17,18^. If the conservation of species with unique combinations of functional traits (i.e. functional distinctiveness) conflates with the maintenance of ecosystem processes and services, then the measure of species’ functional distinctiveness would provide a solid basis for guiding both protection and restoration strategies.

Among single species conservation strategies, reintroduction aims to re-establish a population within the indigenous range of the focal species following local extinction, through the release of a limited number of individuals. The principal objective of reintroduction projects is to improve the conservation status of the focal species, however, restoring lost ecological functions or services may also drive the implementation of population restoration projects^19^, which is a key component of rewilding initiatives^20–22^. In such cases, the primary focus of the translocation shifts from single-species conservation to the inclusion of whole ecosystem management targets. For example, trophic rewilding initiatives aim to restore trophic cascade through the (re)introduction of large species which are expected to have substantial impact at the landscape level through the restoration of top-down interactions that structure ecosystem dynamics^22–24^. Conservation translocations involve a variety of stakeholders with different values, interests and objectives, and priority might be accorded to species based on a variety of criteria^25,26^. Species can be selected based on their ecological role, but general public awareness and political support are also key to ensure a sustainable reintroduction effort^27^. Hence reintroduction initiatives can be promoted by focusing on flagship species (i.e., iconic or charismatic species gathering support and funding), or on species that are valued on grounds of cultural heritage. Motivations behind reintroduction projects are thus likely complex and the incentives are context- and species-specific.

Reintroductions rely on a parochial approach to conserving species^28^ and generally focus on local or national conservation needs^29^, which do not necessarily conflate with conservation priorities at larger scales. Reintroductions are generally case by case initiatives that are not collectively designed to tackle global or continental conservation issues related to the preservation of different facets of biological diversity^30^. The study of the distribution of reintroduction efforts is thus required in order to explore how population restoration strategies may assist the conservation of the diversity of ecological strategies at larger scales. Studies have documented taxonomic biases in reintroduction programs^31,32^, which in turn influence the diversity of evolutionary histories of reintroduction targets^30^. However, how these patterns translate in terms of functional trait diversity has yet to be documented. Reintroduction practitioners could have focused on rare species which may support unique functions, but the relationship between functional distinctiveness and reintroduction efforts remains non-trivial.

Here, we explored the range of functional traits supported by reintroduced terrestrial mammals and birds in Europe, and assessed the extent to which reintroductions may have contributed to the conservation of the European functional diversity for these two groups. We used data on behavioural traits reflecting the way species acquire resources from their environment (feeding behaviour and period of foraging activity), and information on body mass and diet traits which reflect the resource use requirements of species^33^. For each taxonomic group (mammals and birds), we built functional trees to represent the differences in trait values between species in each European assemblage. First, we calculated the functional diversity^14^ of each subset of reintroduced species (i.e., reintroduced birds and reintroduced mammals), that is, the extent of complementarity among species’ trait values, to assess whether a focal subset of reintroduced species is representative of the functional diversity of the regional assemblage. Second, we quantified the functional uniqueness of reintroduced species using the Functional Distinctiveness index^17^, which estimates the conservation value of each individual species based on its unique combination of functional trait values. We constructed null models to test the deviation of our two metrics from the value expected when species were randomly drawn in the associated regional functional tree. The distribution of reintroduction efforts is likely to be driven by patterns of species’ susceptibility to extirpation due to human pressure, which may also be linked to specific trait values such as high body mass^34,35^. If reintroduction programs target some particular groups of species (e.g., raptorial bird species, large ungulates), then we expect the breadth of ecological functions involved, as depicted by the combination of functional traits supported by reintroduced species, to be narrow.

## Methods

### Data and functional trees

We focused on 28 terrestrial mammal species (14% of the 202 European species), and 37 terrestrial breeding bird species (10% of the 378 European species), which have been reintroduced at least once in Europe. Reintroduced species were identified through a comprehensive search of the reintroduction-related literature, and details of the literature research protocol and complete lists of reintroduced species can be found in Thévenin et al.^30^. For functional traits, we used the dataset published by Wilman et al.^36^ who compiled functional trait values for all 9993 and 5400 extant bird and mammal species from the literature. These traits represent how a given organism impacts the community structure and species interaction network, with potential consequences for ecosystem functioning^6,37–39^. They can hence be considered as “effect” traits, which differ from “response” traits, i.e. traits that determine the response of organisms to environmental change^40,41^. These traits are relevant to the “Eltonian niche”, i.e. a multidimensional space describing biotic interactions and resource-consumer dynamics related to the acquisition of energy and nutrients. This dataset provides information on body mass, diet type, foraging behaviour along a vertical gradient (foraging stratum) and period of foraging activity (e.g. nocturnal, diurnal). Body mass is a continuous variable given in grams. A species’ diet is described as a multistate nominal variable representing whether the species’ diet includes one or several of the following eight categories: Invertebrate, Vertebrate, Fish, Carrion, Nectar, Seed, Fruit and Plant (e.g. grass, ground vegetation, seedlings, weeds…). For birds, the foraging stratum is also given as a multistate nominal variable with seven different discrete levels from below the water surface to aerial foraging. For mammals this variable is categorical, with species assigned to only one category (ground level including aquatic foraging; ground foraging; scansorial; arboreal; aerial). Finally, the period of foraging activity is given as a multistate nominal variable for mammals (diurnal, nocturnal and/or crepuscular), while it is a binary variable for birds (nocturnal vs diurnal). Functional trait values for reintroduced birds and mammals are available in Supplementary Dataset.

We built up functional trees from functional traits distances between each pair of species^14^. We calculated pairwise functional distances using a mixed-variable coefficient that allows various types of variables to be included. Euclidean distance was used for body mass (log-transformed), and Gower distance for other types of traits (e.g., multistate nominal). The pairwise distances were calculated using the *dist.ktab* function in the *ade4* R-package. We then applied hierarchical classification methods to synthetize the multidimensional trait space into a functional tree. Representing a functional multidimensional space (each functional trait being an axis of this space) using a dendrogram can result in a loss of information because the distances between species are based on the lengths of the branch connecting the tips in the functional tree (i.e. the cophenetic distances), instead of the aggregation of the pairwise distance on each trait in multidimensional functional space. Following Mouchet et al.^42^, we selected the clustering method which led to the lowest amount of distortion between the initial and cophenetic pairwise distance matrix. The Unweighted Pair Group Method with Arithmetic Mean (UPGMA) provided the most robust functional trees, which is consistent with results from other studies^43^. Maire et al.^44^ proposed a framework for evaluating the quality of a functional trait space based on the average deviation between the initial distances calculated with trait values and the cophenetic distances between species in the functional trait space^44^. They showed that in some cases functional dendrograms might artificially increase functional distances between pairs of species, and that this bias depends on the number and types of traits considered. To test for the quality of our functional dendrograms, we computed Maire’s “mSD” metric that measures the average squared deviation between the initial Gower’s distances and the cophenetic distances on the functional dendrograms for both mammals and birds. In both cases, the deviation was below the 0.01 threshold value used by Villéger et al.^45^ to determine the quality of the functional space^45^ (mSD[birds] = 0.008; mSD[mammals] = 0.005]). Thus, we consider that our dendrogram-based approach is a reliable representation of the initial functional trait values, and appropriate to investigate functional trait diversity patterns for reintroduced targets in Europe.

### Functional diversity of reintroduced species

Functional Diversity (hereafter FD) measures the complementarity among species’ trait values in a particular species assemblage through the estimation of the dispersion of species in trait space. Given one functional tree, the functional diversity of a subset of species is measured as the sum of the length of the branches in the minimal subtree connecting all taxa of the subset:

1 $$FD\left( {tree} \right) = \mathop \sum \limits_{j} L_{j}$$

with *L*_*j*_ representing the length of branch *j*. For a given subset of species, the higher the FD, the more functionally dissimilar the species are within the subset. For each taxonomic group (terrestrial mammals and terrestrial birds), we calculated the FD of the subset of reintroduced species [FD_reint_] using the *pd.query* function from the *PhyloMeasures* R-package^46^. For each taxonomic group, we compared this value to the FD value expected for a random subset of species of the same size in the associate regional species pool. For that purpose, we used the pd.moments function (Phylomeasures package), which provides optimized algorithms to compute the exact expressions of the expectation [*µ*FD] and the SD[*sd*FD] of the FD for a given number of species in a functional tree. We considered that a subset of reintroduced species was representative of the regional functional diversity if the FD_reint_ value did not significantly depart from the associated 95% confidence interval calculated as *µ*FD ± 1.96*sdFD.

### Functional distinctiveness of reintroduced species

We measured the functional uniqueness of individual species using the Functional Distinctiveness index (hereafter, FDist)^17^, which is based on the fair-proportion index^47^. Given a functional tree, the FDist score of the species *i* is the total branch length between each node connecting the tip (species) to the root of the tree, each time divided by the number of species subtending that branch:

2 $$FDist_{i} = \mathop \sum \limits_{{j \in P\left( {i, Root} \right)}} \frac{{L_{j} }}{{n_{j} }}$$

with *P*(*i, Root*) being the set of branches connecting species i to the root of the tree, and *n*_*j*_ being the number of species subtending branch *j*. The FDist scores of all species in a subset sum up to the Functional Diversity of the whole subset (sum of the lengths of the branches of a given functional tree sensu Petchey and Gaston^14^). We used the *evol.distinct* function from the *ape* package^48^ to calculate the FDist scores for terrestrial mammals and terrestrial birds in each European assemblage. We assessed whether reintroduced species were more or less functionally distinct than expected if species were randomly drawn from the regional species pool. For each taxonomic group (terrestrial mammals and terrestrial birds), we used the median FDist of the subset of reintroduced species rather than the mean given the skewness of the distribution of FDist scores, and compared the median FDist to the 95% confidence interval of the null distribution obtained by drawing 10,000 random samples of species of the same size in the associated regional functional tree. The departure from the expected median FDist produced by our null model was expressed as a p-value and was calculated as the number of random median FDist values that were superior to the median ED of reintroduced species and divided by the number of randomly drawn subsets.

## Results

**Figure 1:**
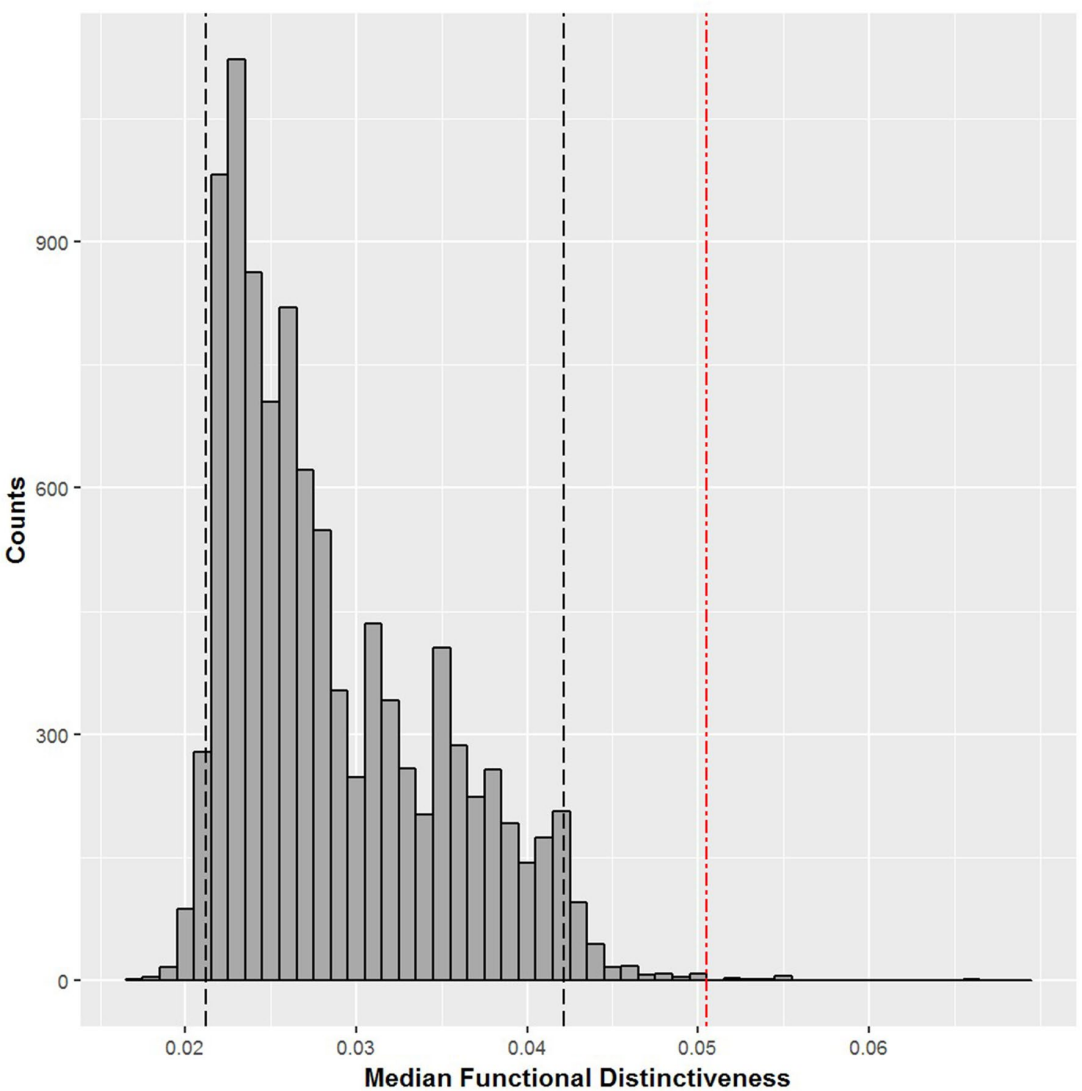
The null distribution of the median Functional Distinctiveness for 28 mammal species randomly drawn from the functional tree of European terrestrial mammals (10,000 samples). Black dashed lines represent the 95% CI interval (i.e. [0.0212, 0.0421]), and the red dashed line represent the observed median FDist value for reintroduced mammals (median FDist_reint_ = 0.0505, p-value < 0.001).

We found that the diversity of trait values combinations carried by reintroduced species of mammals in Europe is not highly representative of the functional diversity of the continental assemblage. The functional diversity (FD) of the 28 reintroduced species of mammals was lower than expected under our null model (FD_reint_ = 3.857, *µ*FD = 4.807, *sd*FD = 0.443, p-value = 0.03) (Table 1). However, our analysis showed that several reintroduced species are among the most functionally distinct species of the terrestrial mammal assemblage in Europe (Supplementary Dataset). Thus, despite taxonomic biases being associated with relatively low functional diversity, our analysis showed that the median FDist of the 28 reintroduced mammals is higher than expected under our null model (median FDist_reint_ = 0.0505, p-value < 0.001) (Fig. 1). The most functionally distinct reintroduced mammal in our data is the European pine marten (*Martes martes*, FDist = 0.2388, rank = 4 out of 202 European mammal species). The other highly functionally distinct reintroduced mammals are the edible dormouse (*Glis glis*, FDist = 0.2065, rank = 9/202), the hazel dormouse (*Muscardinus avellanarius*, FDist = 0.1893, rank = 13/202) and the red squirrel (*Sciurus vulgaris*, FDist = 0.1884, rank = 14/202).

**Table 1:** Functional diversity (FD) for each subset of reintroduced birds and mammals in Europe, and the associated expected value µFD and sdFD for the associated subset size (number of reintroduced species in each group) under our null model. Deviation from the null model is presented as a p-value from a Z-test statistics. Bold values indicate p-value < 0.05.

GROUP	No. of native terrestrial species	No. of reintroduced species	FD of reintroduced species	Expected FD	SD of FD	*p*-value
Mammals	202	28	3.857	4.807	0.443	**0.03**
Birds	378	37	8.364	8.611	0.458	0.59

**Figure 2:**
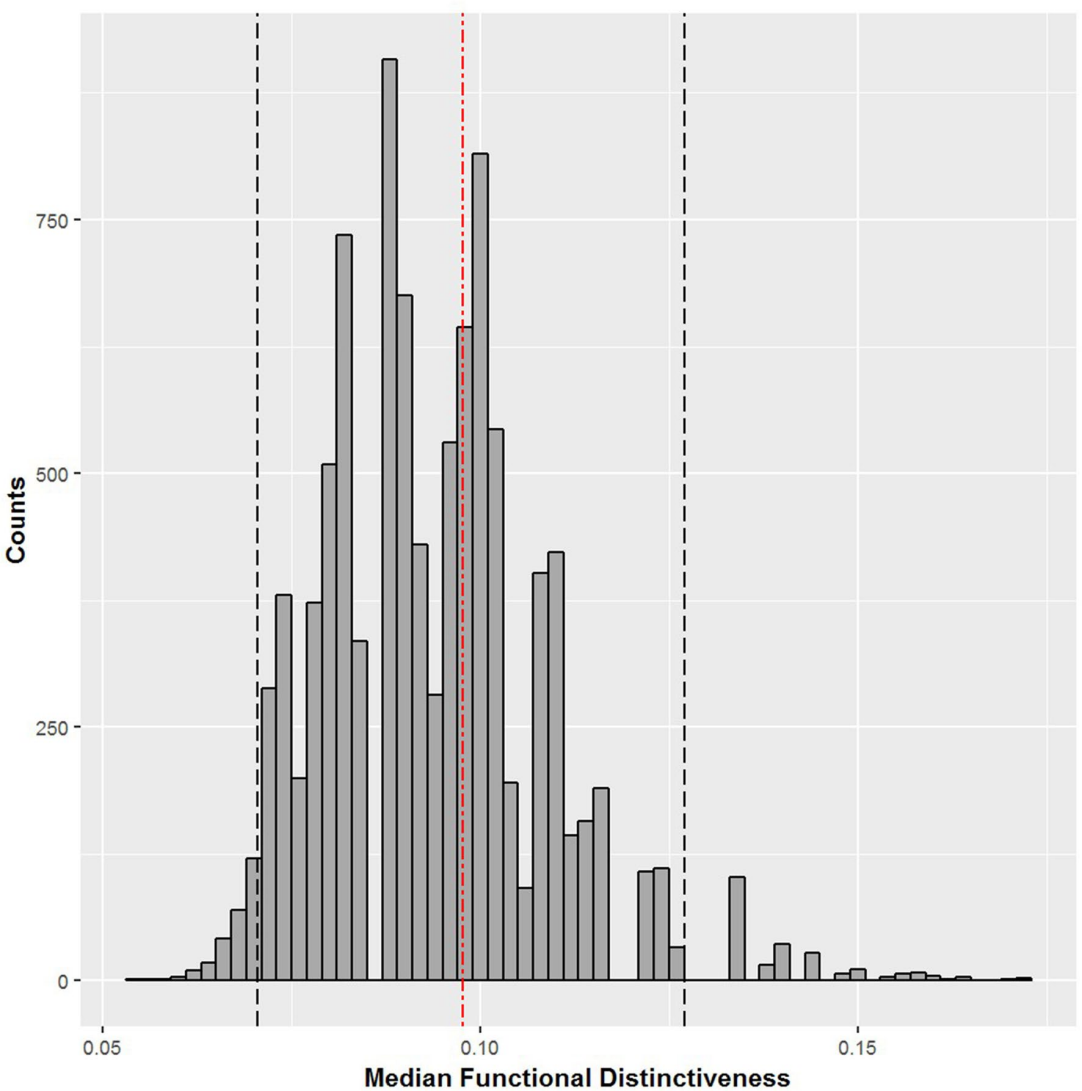
The null distribution of the median Functional Distinctiveness for 37 bird species randomly drawn from the functional tree of European terrestrial breeding birds (10,000 samples). Black dashed lines represent the 95% CI interval ([0.0715, 0.1331]), and the red dashed line represent the median FD value for reintroduced birds (median FD_reint_ = 0.0976, p-value = 0.79).

Unlike mammals, we found that reintroduced birds are collectively representative of the functional diversity of the European avian assemblage. Our results show that there is no significant deviation from the distribution of FD values expected if reintroduced bird species were randomly sampled from the continental assemblage of terrestrial breeding birds (FD_reint_ = 8.364, *µ*FD = 8.611, *sd*FD = 0.458, p-value = 0.59) (Table 1). Reintroductions of birds have involved a few species that are highly functionally distinct at the continental scale. The most functionally distinct reintroduced bird is the common raven (*Corvus corax*, FDist = 0.2807, rank = 5 out of 378 European terrestrial bird species) with its highly diverse diet comprising almost all categories except nectar, followed by the Osprey (*Pandion haliaetus*, FDist = 0.2552, rank = 14 out of 378 European terrestrial bird species) with its specialized piscivorous diet. However, unlike mammals, reintroduced birds are not more functionally distinct than expected, and the median FDist of reintroduced birds was close to the random expectation (median FD_reint_ = 0.0976, p-value = 0.79) (Fig. 2).

## Discussion

Functional diversity has been advocated as a biodiversity measure that accounts for dissimilarities in species’ forms and functions, and here we provide new insights on how reintroduction targets can be representative of the diversity of a continental assemblage of species and thus contribute to the conservation of biological diversity at large scales. The data we collected show that reintroductions of birds in Europe are taxonomically biased and mainly involve raptorial and game species. Interestingly, although these taxonomic biases are associated with less phylogenetic complementarity for reintroduced birds at the European scale^30^, our results show that reintroductions of terrestrial birds in Europe targeted diverse combinations of functional traits. Our findings also indicate that reintroduction programs involved species of terrestrial mammals in Europe that are individually more functionally distinct than if drawn at random from the continental assemblage, but that, collectively, these species carry less functional diversity than expected. The arboreal/scansorial foraging stratum, coupled with diverse diets seem to explain the higher functional distinctiveness for mammals.

Functional trait diversity provides a mechanistic framework to assess the ecological roles of species, but the outputs may be more meaningful when considering interacting species that share the same environmental conditions and resources or that co-evolved under similar biogeographical regions and historical processes^49^. These conditions might not be met in our study because of the continental spatial scale, which does not consider the presence or absence of spatial overlap between species. Depending on the relative spatial distributions of species, some species might not appear as ecologically distinct in the continental pool of species, although they are ecologically distinct at a local scale, and thus play an important functional role locally. Considering differences in functional trait values at the scale of continental assemblages comes with some limitations, however the species supporting the most distinct associations of functional traits at such large scale will likely remain among the most functionally distinct species wherever they might occur. In addition to identifying which reintroduced species are functionally distinct at large scale, we need to locate where they might also be distinct at the local scale by considering where the reintroduction has been implemented (release site) and assess the local assemblage of species with which the reintroduction target is likely to interact.

Here we used functional dendrograms and the fair-proportion index^17,47^, and functionally distinct species are those that contribute to the functional diversity of the assemblage because they support a combination of trait values (diet, activity, body mass and foraging height) that is not supported by other species on the functional tree. The extent to which such distinctiveness relates to key ecological processes depend on the types of traits used, and how trait complementarity contribute to ecosystem functioning^50–52^. Moreover, the continuum between functionally distinct and functionally redundant species is not straightforwardly consistent with other important concepts in conservation such as the continuum between specialist and generalist species^53^ or ecosystem engineers^54,55^. Therefore the question of whether reintroduction practitioners have focused on functionally distinct species might not reflect the fact that ecological processes are given increased attention in the reintroduction literature and practice^56,57^. For example, three out of the four European species of vultures, i.e. the only obligate scavengers among vertebrates, have been reintroduced in Europe. Among other aspects, incentives for reintroducing large vultures are based on their specialized scavenger diet^58,59^. Here, the fact that these four species share similar trait values and are considered altogether in the same continental pool of species led to reintroduced vultures not being particularly functionally distinct.

The study of functional diversity allows to explore the value and dissimilarity of morphological, ecological and behavioural traits in biological assemblages, but its ability to describe a species ecological function is highly constrained by the type and number of traits considered^44,60^. The type of functional traits considered, and the way they are weighted in calculating species’ dissimilarities largely influence the measure of functional diversity and species’ rankings based on functional distinctiveness. Our analysis showed that, when considering the whole continental assemblage of European terrestrial mammals, large herbivores are not particularly functionally distinct, because all members of the Artiodactyla order in Europe (except the wild boar, *Sus scrofa*) share the same diet types (plant material), foraging strategy (ground feeder) and, to some extent, have similarly large body masses. Our data show that reintroduction projects within mammals have involved many ungulates, but an analysis at the continental scale will consider these associations of functional trait as relatively redundant, as these species differ only when considering their period of activity. Here the lack of accuracy in traits discrimination, e.g. ignoring the different types of grazing or browsing among ungulates, may partly affect this result. The functional differences described here mostly concern species’ resource use patterns. Resource use may not reflect finer divisions in some functional groups and may be less appropriate to accurately describe some ecosystem processes. It may thus overlook the important role of some individual species^61^. While the idea of prioritizing species based on their functional originality is promising, it remains challenging in practice and enhancements of trait databases are needed for vertebrates. Information on functional traits has been made increasingly available for animals but the number of traits considered, and the extent to which they relate to an ecological function is still limited compared to plants^40,62^. In the dataset we used, diet types and body mass provided a proxy for the trophic level of a species, but will mostly help differentiate herbivores from carnivores. One major element that could be integrated in such analyses is information on the type and number of species’ trophic interactions.

Alongside the improvement of the conservation status of the focal species, reintroductions provide opportunities to restore lost ecological functions and processes in degraded ecosystems^19^, and the study of functional diversity patterns could play a substantial role in reintroduction planning. Before implementing releases, managers are advised to conduct feasibility studies and assess the potential risks associated with translocating the focal species. These risks can be sociological (e.g. Human-wildlife conflicts associated with the reintroduction of apex predators), but also ecological because of the potential deleterious effects associated with the re-integration of a species in a trophic network or, more broadly, on other species in the ecosystem. Some systems may have undergone profound changes in community composition, depending on the time elapsed between the extirpation and the return of the species^63,64^. Feasibility studies must address biotic interactions (competition, predation), to predict the impact of the return of the focal species to the community, which could have undergone significant ecological and evolutionary changes since extirpation^65^. However, managers may lack the tools to do so, and trait-based approaches could help foreseeing these negative impacts, both for the focal species, but also for the recipient community. Such approaches could contribute to identify reintroduction targets that will enhance functional complementarity at the scale of the communities and, hopefully, improve ecosystem functioning. Unfulfilled functional roles can be viewed as opportunities for species translocation, and in some cases population restoration projects may improve both the conservation status of the focal species along with the functioning and resilience of restored ecosystems^66^. This is an increasingly used argument in rewilding projects that incorporate the reintroduction of locally extinct species or, in some cases, their ecological replacement with surrogates based on their ability of restore lost functions in ecosystems^19,22,67^. The study of functional diversity patterns is increasingly used to assess the impact of conservation efforts^68,69^, and therefore recommendations are needed to further integrate species-based and trait-based approaches so that they can mutually enrich each other. For example, trait-based approaches could help foreseeing negative ecological impacts, both for the focal species, but also for the recipient community. On the other hand, reintroductions can also represent a way to experiment at large scale in ecology^70^, and reintroduction and rewilding experiments could be used to improve our understanding of the impact of functionally distinct species in natural communities, or investigate competition in niche dimensions induced by the return of the focal species.

## Supplementary Information

Dataset S1.
